# Implementation and effectiveness of a nurse-enabled, shared-care follow-up model for early breast cancer survivors (The IBIS-Survivorship Study): protocol for a stepped-wedge cluster randomised trial

**DOI:** 10.1136/bmjopen-2025-103341

**Published:** 2025-06-19

**Authors:** Raymond J Chan, Fiona Crawford-Williams, Bogda Koczwara, G Bruce Mann, Elizabeth Eakin, Jane Turner, Meinir Krishnasamy, Louisa G Collins, Helen Skerman, Karla Hemming, Nicolas H Hart, Jon Emery, Bethany Crowe, Kerry Patford, Jane Mahony, Caroline Kristunas, Gillian Blanchard, Laura Healey, Jasotha Sanmugarajah, Rhea Liang, Paul Craft, Amy Shorthouse, Ruth McCaffery, Mary Azer, Patsy Yates

**Affiliations:** 1 Caring Futures Institute, Flinders University, Adelaide, South Australia, Australia; 2 Cancer and Palliative Care Outcomes Centre, Queensland University of Technology, Brisbane, Queensland, Australia; 3 Princess Alexandra Hospital Division of Cancer Services, Woolloongabba, Queensland, Australia; 4 McGrath Foundation, Sydney, New South Wales, Australia; 5 Flinders University School of Medicine, Adelaide, American Samoa; 6 The Royal Melbourne Hospital, Melbourne, Victoria, Australia; 7 The Royal Women’s Hospital, Parkville, Victoria, Australia; 8 Australia and New Zealand Breast Cancer Trials Group, Newcastle, New South Wales, Australia; 9 The University of Melbourne Department of Surgery, Melbourne, Victoria, Australia; 10 The University of Queensland School of Human Movement and Nutrition Sciences, Brisbane, Queensland, Australia; 11 The University of Melbourne The Sir Peter MacCallum Department of Oncology, Melbourne, Victoria, Australia; 12 Academic Nursing Unit, Peter MacCallum Cancer Centre, Melbourne, Australian Capital Territory, Australia; 13 VCCC Alliance, Parkville, Victoria, Australia; 14 Cancer Council Queensland, Spring Hill, Queensland, Australia; 15 Population Health Program, QIMR Berghofer Medical Research Institute, Brisbane, Queensland, Australia; 16 School of Nursing, Queensland University of Technology, Brisbane, Queensland, Australia; 17 Institute of Applied Health Research, University of Birmingham, Birmingham, England, UK; 18 INSIGHT Research Institute, University of Technology Sydney, Sydney, New South Wales, Australia; 19 Edith Cowan University Exercise Medicine Research Institute, Joondalup, Western Australia, Australia; 20 Department of General Practice and Primary Care, University of Melbourne, Melbourne, Victoria, Australia; 21 The University of Melbourne Centre for Cancer Research, Parkville, Victoria, Australia; 22 University of Birmingham, Birmingham, UK; 23 Calvary Mater Newcastle, Waratah, New South Wales, Australia; 24 School of Nursing and Midwifery, The University of Newcastle, Newcastle, New South Wales, Australia; 25 School of Medicine, Griffith University, Southport, Queensland, Australia; 26 Gold Coast Hospital and Health Service, Southport, Queensland, Australia; 27 Bond University, Gold Coast, Queensland, Australia; 28 Medical Oncology, Australian National University Medical School, Canberra, Australian Capital Territory, Australia; 29 Canberra Hospital, Woden, Australian Capital Territory, Australia; 30 West Moreton Hospital and Health Service, Ipswich, Queensland, Australia; 31 Sunshine Coast University Hospital, Birtinya, Queensland, Australia; 32 School of Nursing, Queensland University of Technology, Kelvin Grove, Queensland, Australia

**Keywords:** Primary Care, Breast tumours, Nursing Care

## Abstract

**Introduction:**

Breast cancer is the most commonly diagnosed cancer among women worldwide. Survivors often experience physical and psychological effects arising from breast cancer and its treatment, which can last months and years, adversely impacting quality of life. As the number of early breast cancer survivors increases, models of specialist-led follow-up care in hospital settings are not sustainable and evidence suggests that they may not meet survivors’ needs. Nurse-enabled, shared-care, follow-up models between cancer specialist and primary care teams have potential to address this need.

**Methods and analysis:**

The proposed research is a multicentre, prospective, pragmatic, stepped-wedge cluster-randomised trial designed to test the effectiveness and implementation of *IBIS-Survivorship*, a follow-up care model for patients with early breast cancer who have completed primary treatment. The *IBIS-Survivorship* intervention involves a nurse-led consultation, development of a Survivorship Care Plan and case-conferencing between a breast care nurse and the patient’s primary care provider. This study seeks to recruit 1079 breast cancer survivors across six cancer centres (clusters) in Australia. Health-related quality of life at 12 months assessed by the Functional Assessment of Cancer Therapy - Breast Cancer questionnaire will be the primary endpoint, along with a range of patient-reported outcomes, safety indicators and cost-effectiveness measures as secondary endpoints. General and generalised linear mixed models will be used to assess the effectiveness of the intervention versus usual care. Implementation and process outcomes will be assessed using the Reach Effectiveness Adoption Implementation Maintenance framework.

**Ethics and dissemination:**

Ethical approval was provided by the Metro South Hospital and Health Service Human Research Ethics Committee (HREC/2020/QMS/59892) and reciprocally across the other five trial sites under National Mutual Acceptance arrangements. Results will be disseminated through peer-reviewed academic journal publications and presentations at national and international conferences.

**Trial registration:**

Australia and New Zealand Clinical Trials Registry (ANZCTR) Trial ID: ACTRN12621000188831.

**STRENGTHS AND LIMITATIONS OF THIS STUDY**

- The IBIS-Survivorship model is a complex intervention intended to take a coordinated approach to implementing shared care follow-up that can be tailored to the needs of individual patients.
- The hybrid effectiveness-implementation design will evaluate the IBIS-Survivorship shared care follow-up model in diverse breast cancer clinic settings.
- Each cluster has different practicalities in follow-up care to suit their unique setting. A strength of using the stepped-wedge cluster-randomised trial design allows for the evaluation of service delivery without contamination between intervention and control clusters.
- The study was limited to six clusters due to practical constraints.
- Collection of patient-reported outcomes, health service utilisation and qualitative data will gather extensive insights into both effectiveness and implementation outcomes.

## Introduction

 Breast cancer is the most commonly diagnosed cancer in Australian women and worldwide, with 5-year survival rates in Australia at 92%.^1 2^ This high survival rate means there is a growing number of people requiring ongoing care after treatment completion for breast cancer to manage a variety of long-term biopsychosocial effects from the cancer diagnosis and treatment.^3^ Furthermore, many cancer survivors, including those diagnosed with breast cancer, have comorbid conditions that require ongoing management.^4^ Cancer survivors are more likely to develop circulatory, musculoskeletal and endocrine system disorders and psychological and behavioural problems compared with those without a cancer diagnosis.^4^ The breadth of these concerns accentuates the need for seamless, multidisciplinary, patient-centred care following the completion of primary breast cancer treatment in order to comprehensively address patient needs.^5^ In Australia, predominant models of post-treatment follow-up care for breast cancer are hospital-based and driven by oncology specialists, focusing on surveillance for disease recurrence rather than addressing personalised survivorship care needs. Such models are not sustainable and limit coordination of care between oncology specialist services and primary care.^6 7^ With an ever-growing population of breast cancer survivors, there is a need for a coordinated pathway of post-treatment care involving oncology specialists and primary care teams.^8^

A shared care model for early breast cancer follow-up is consistent with Australian policies including Cancer Australia guidelines^9^ and the Optimal Care Pathway for breast cancer,^5^ as well as international recommendations.^10^ Shared care is follow-up after treatment completion that is shared between a hospital-based specialist team and primary care provider, usually a general practitioner (GP—Australian primary care physician) where both parties have ongoing involvement in the patient’s care. For many cancer survivors, such a model has been demonstrated to be safe, effective, acceptable, cost-efficient and more patient-centred.^11 12^ For example, a Canadian multicentred, randomised controlled trial of patients who completed treatment for early breast cancer (n=968) concluded that follow-up care led by primary care providers was safe and there were no differences in recurrence-related serious clinical events.^13^ Similar Australian studies have concluded that shared care between oncology specialists and GPs is feasible, acceptable and as safe as specialist-led models.^14 15^ Importantly, shared care models demonstrate similar effectiveness to usual care (ie, specialist-led care) in terms of patient quality of life.^1115^,^17^ GPs are well placed to address psychosocial issues, promote healthy lifestyle behaviours and address comorbid health conditions compared with oncology specialists.^14^ Additionally, an economic modelling study indicated that shared care is more cost-effective than specialist-led care and could save over $A3.5 million for the 500 patients in this study over 5 years by releasing specialist time for more urgent cases, relieving pressure on the acute care system.^14^

Although the evidence for shared care models in early breast cancer is promising, these models are not routinely implemented into practice in Australia, as barriers such as lack of role clarity, inadequate support to transfer care and information across acute care and primary care systems and communication difficulties impact on successful implementation.^18^ Research has investigated the role of nurse-led models of survivorship care, highlighting that nurse-led care can improve quality of life^19^ and produce cost savings to cancer survivors and healthcare systems.^8^ Specialist cancer nurses in the hospital setting (experienced registered nurses who have received additional training to make them an expert nurse in cancer care) are well-placed to facilitate effective and timely care and communicate with GPs in a shared care model.^20 21^ In Australia, BCNs have provided information and support to patients, as well as co-ordinating patient care, since the ‘90s,^22^ with access to these nurses resulting in an improved experience for patients and families.^23^ Therefore, the opportunity exists for BCNs to contribute further to shared care planning and follow-up care.^23^

The aim of this study is to assess both the effectiveness and implementation of a nurse-enabled shared care follow-up model (*IBIS-Survivorship*) for cancer survivors who have completed primary treatment for early breast cancer in Australia via a stepped-wedge cluster randomised trial (SW-CRT). To our knowledge, there are no SW-CRTs in shared care for patients with early breast cancer that evaluate the effectiveness and implementation of a shared care model of follow-up.

## Methods and analysis

This study protocol (V.2.4) was written in accordance with the Standard Protocol Items: Recommendations for Interventional Trials guidelines^24^ (see online supplemental material A).

The proposed study will use a SW-CRT design to deliver the *IBIS-Survivorship* model sequentially in six acute care cancer centres across metropolitan and rural Australia. *IBIS-Survivorship* is a multicentre, prospective, pragmatic type II hybrid effectiveness-implementation trial of a nurse-enabled shared care model. Our primary hypothesis is that participants receiving the *IBIS-Survivorship* intervention will have a significantly better breast cancer-specific health-related quality of life (HRQoL) at 12 months post enrolment, compared with those receiving usual care. Secondary hypotheses are that participants receiving *IBIS-Survivorship* will have higher satisfaction with care; higher uptake of healthy lifestyle behaviours and equivalent adherence to annual mammography, annual physical examination and endocrine therapy at 12 months post enrolment. It is, however, acknowledged that the collection of these adherence outcomes for the longer term will require additional resources to follow patients. We also hypothesise that *IBIS-Survivorship* will be more cost-effective compared with usual care. Implementation and process outcomes will be assessed using the Reach Effectiveness Adoption Implementation Maintenance (RE-AIM) Framework.^25^

### Study population and study sites

The target population is adults aged 18 years or older diagnosed with early breast cancer as defined by a stage I-III diagnosis (inclusive of ductal carcinoma in situ and locally advanced breast cancer but no distant metastases); within 10 weeks of completion of adjuvant cancer treatment or surgery (if not scheduled for further adjuvant therapy) at a participating cancer centre. Participants will be eligible if they are able to identify a usual GP or GP practice, are ambulatory with an Eastern Cooperative Oncology Group performance status of 0–2 and are able to read and understand English. Cancer survivors will be excluded if they have metastatic breast cancer or the presence of severe mental, cognitive or physical conditions that would limit their ability to participate (as determined by their treating clinicians). Recurrent or secondary primary breast cancer, comorbidities, other prior cancer diagnoses or treatment of trastuzumab (or biosimilars), neoadjuvant and/or ongoing endocrine therapies are *not* exclusion criteria.

The participating cancer centres (clusters) include the Princess Alexandra Hospital (Brisbane, Queensland), Sunshine Coast Hospital and Health Service (Sunshine Coast, Queensland), Gold Coast Hospital and Health Service (Gold Coast, Queensland), Ipswich Hospital (Ipswich, Queensland), Calvary Mater Newcastle (Newcastle, New South Wales) and Canberra Hospital (Canberra, Australian Capital Territory). These centres are large government-funded hospitals that are expected to treat ≥200 people with early breast cancer each year and currently employ at least one breast cancer specialist nurse (BCN). The study opened at all sites in May 2021 and recruitment closed in November 2023. Data collection is ongoing, expected to be finalised in November 2025.

### Study design

**Figure 1:**
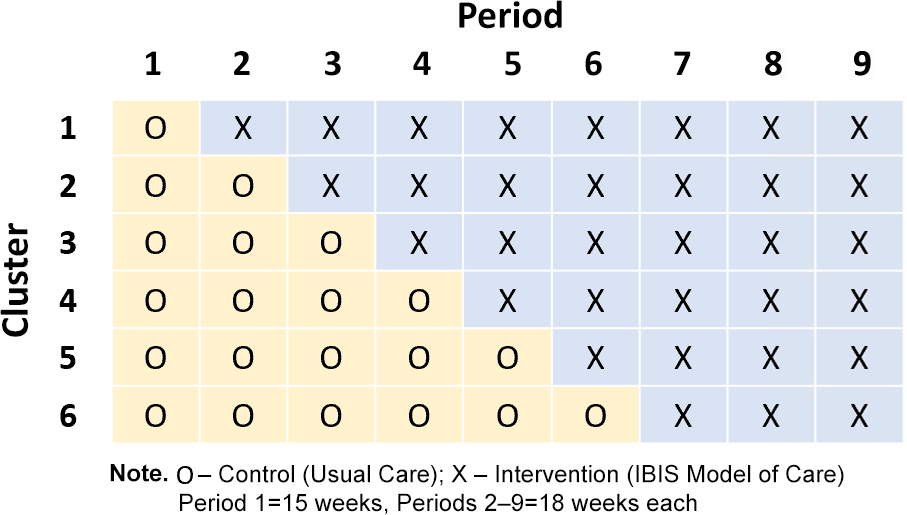
Stepped wedge design schema.

The study will employ a SW-CRT design that involves the sequential rollout of the *IBIS-Survivorship* intervention to each of the clusters in a predetermined, randomly allocated order within the study period. Each sequence of treatments per cluster comprises nine cluster periods (see figure 1). In each cluster period (time of measurement), assessments are collected for each cluster and treatment received. All clusters commence in the usual care condition (standard cancer follow-up care), then every 18 weeks (cluster period) one cluster will crossover from usual care to implementation of the *IBIS-Survivorship* intervention. In each cluster, participants who are enrolled in the study prior to the crossover date will be in the usual care arm and will continue to receive usual care. There is no formal transition period, so participants will continue to be enrolled to clusters and receive usual care during the last 8 weeks of that cluster period until the crossover date. Once a cluster has crossed over to the intervention arm, participants enrolled in the study in the following cluster periods will receive the intervention. Thus, participants will not be exposed to both treatment conditions. Enrolment will stop at the end of cluster period seven when all clusters are in the intervention arm. The study intervention delivery will continue for a further two cluster periods (36 weeks) and will formally end when outcome data are collected from the last enrolled participants. The SW-CRT design allows for a staged implementation process which is helpful when simultaneous rollout of an intervention to all clusters is impractical due to logistical and financial constraints.^26^

### Participant screening and consent

Research nurses will carry out initial screening to identify potential participants. Potential participants will then be reviewed according to the eligibility criteria by a member of the treating team, and if suitable, asked if they agree to be approached by a research nurse for consent to the study. These potential participants will be provided with a brochure giving detailed information about the study objectives and a Participant Information and Consent Form (PICF) providing details of the study assessments and procedures (online supplemental material B). Participants will be given up to 2 weeks to consider their participation. Participants will provide written informed consent to be enrolled into the trial. All participants will be asked to consent to allow the study team access to their medical record, and a separate written consent form will be used to indicate the participant’s willingness to allow for access to Australian Medical Benefits Schedule and Pharmaceutical Benefits Scheme (PBS) data through Services Australia.

Once a participant consents, this will be recorded in a REDcap^27 28^ database and they will be assigned a unique study ID number. Participants will be automatically invited to complete a baseline survey (T1) on enrolment. This provides sociodemographic information, medical/health history and baseline measures for patient-reported outcomes. If they do not complete this survey within 10 weeks of completion of adjuvant cancer therapies or surgery, the participant will be withdrawn from the study.

### Intervention

**Figure 2:**
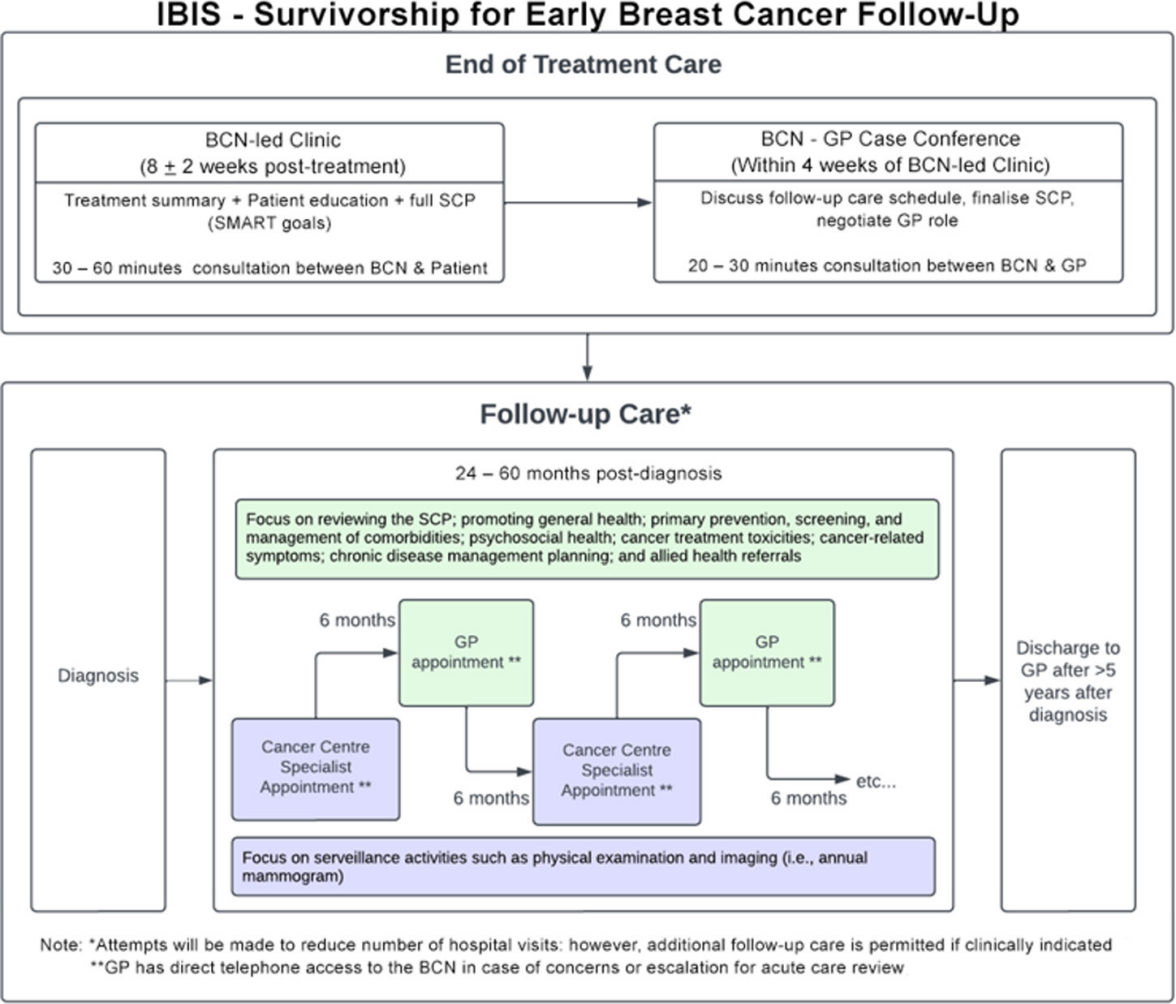
IBIS-Survivorship intervention model. BCN, Breast Cancer Nurse; SCP, Survivorship Care Plan; GP, General Practitioner; Phy Exam, Physical Examination.

The *IBIS-Survivorship* model of care is a multifaceted intervention that includes a prespecified shared care pathway between oncology specialists and GPs following completion of primary cancer treatment. table 1 outlines the three key activities that comprise the intervention and figure 2 outlines the expected *IBIS-Survivorship* shared care pathway. Activity 1: within 6 weeks to 10 weeks after the completion of active treatment, a 30–60 min consultation between the participant and the BCN is conducted to discuss treatment, provide education and to develop a Survivorship Care Plan (SCP). The SCP will include goal setting of up to three SMART (specific, measurable, achievable, realistic and timely) goals that are developed in partnership between the participant and the BCN, using motivational interviewing and self-efficacy techniques. The initial SCP will be sent to the GP and the participant within 3 days of this consultation. Activity 2: within 4 weeks of the nurse-led consultation, a 20–30 min case conference between the BCN and the participant’s nominated GP is completed to discuss the shared follow-up care schedule, discuss the SCP and negotiate the GP’s role in facilitating the SCP goals. The GP may propose changes or express if they are not willing to take part in specific care activities outlined in the SCP. If a formal case conference is not achievable, a short phone call between the BCN and GP will occur, and if no contact with the GP can be made, a copy of the SCP will be sent to the GP practice records. Activity 3: the participant attends a GP appointment to discuss the SCP, and the shared care follow-up schedule commences consisting of 6 monthly appointments with the oncology specialist team and annual appointments with the GP for up to 5 years post-diagnosis. The GP will be provided with direct telephone access to the BCN in case of concerns or escalation for acute care review. At 5 years, the patient will be discharged to the care of the GP.

**Table 1:** Active ingredients of the IBIS-Survivorship intervention model

Active ingredient	Personnel involved	Specific activities	Note
BCN-led consultation providing treatment summary and survivorship care planning (SCP; 30–60 min)	BCN, participant	Treatment summary, co-draft SCP, collaborative planning for health goals, post-treatment education, motivational interviewing and assessment of self-efficacy.	The completed treatment summary and draft SCP will be sent to GP within 1 week after this BCN-led clinic. The BCN will organise the case conferencing with the GP.
Case conferencing with GP (duration variable pending GP availability and need)	BCN, GP	BCN to present the treatment summary and SCP. BCN to negotiate follow-up responsibilities with the GP and respond to GP queries.	Draft SCP will be sent to the GP and the participant. A final copy will be added to the medical record.
Standardised shared follow-up care between oncology specialists and GPs (duration variable pending need)	Oncology specialist(s)*, GP, patient	The oncology specialist(s) to review the patient, order and review the mammogram and undertake a full physical examination every 12 months.The GP to review the patient at least every 12 months to carry out SCP-specific care activities†	The GP and the participant have the direct telephone number of the relevant BCN for advice or to escalate interventions to return to acute care for review.

* Oncology specialists can refer to surgeons, medical oncologists and radiation oncologists.

† Activities may include, but are not limited to, general health and comorbidity management, psychosocial screening and health management, management of cancer treatment toxicities and cancer-related symptoms; chronic disease management planning and subsequent allied health referrals.

BCN, Breast Cancer Nurse; GP, General Practitioner; SCP, Survivorship Care Plan.

The design of *IBIS-Survivorship* is informed by a number of Cancer Australia guidelines,^29^ the Optimal Care Pathway,^5^ Bandura’s self-efficacy model^30 31^ and our extensive pilot work including a systematic review^32^ and observational studies.^33^,^36^ Training for the BCNs involved in delivering the intervention will be standardised using strategies previously tested by the research team. All BCNs delivering *IBIS-Survivorship* will meet the minimum standard required for specialist cancer nursing practice as set out in the competency standards from Cancer Australia.^37^ The BCNs will be required to complete an online Cancer Survivorship Introductory Course (approximately 4.5 hours over six modules: https://education.eviq.org.au/courses/supportive-care/cancer-survivorship), undergo 3 hours of interactive training on intervention delivery (including motivational interviewing and assessment of self-efficacy) designed by an experienced psycho-oncologist (JT) and undergo role-play preparation with the research team. This training will be delivered prior to crossover to the intervention. BCNs delivering the intervention will also receive clinical supervision for 12 months after notification of the intervention crossover date from a member of the research team with clinical expertise in breast cancer care (KP). Due to the nature of the pragmatic trial, it is expected that the delivery of *IBIS-Survivorship* will allow for variation based on the clinical presentation of each individual participant.

### Control arm

Participants who are enrolled in the control arm will receive usual care or the support they usually receive from their healthcare teams. The current follow-up arrangement in all participating clusters is a specialist-led model as determined by the individual treating surgeons, medical oncologists or radiation oncologists. Usual care may differ at each site. All participants will be provided with a written survivorship booklet ‘Living Well After Cancer’ published by Cancer Council Australia^38^ regardless of arm assignment.

### Outcomes

#### Effectiveness outcomes

##### Primary endpoint

HRQoL as measured by the Functional Assessment of Cancer Therapy - Breast Cancer (FACT-B)^39^ at 12 months post enrolment is the primary outcome measure. This validated and reliable tool is well accepted for use in cancer survivors undergoing and beyond treatment^40^ and captures key domains of QoL and key symptoms that are relevant to the study population and sensitive to changes over time.

##### Secondary endpoints

The secondary endpoints include a range of quantitative patient-reported outcomes at 6 months and 12 months post-enrolment (T2, T3) outlined in table 2. Furthermore, open-ended responses exploring participant satisfaction with the *IBIS-Survivorship* model will be collected through the T3 survey.

**Table 2:** Measurement and data collection schedule

Setting	RE-AIM indicator	Collection method/ tools	Study period			
			T1	T2	T3	End of study
*Primary outcome*						
Patient	Effectiveness					
	Health-related quality of life (HRQoL)	Self-report: Functional Assessment of Cancer Therapy - Breast Cancer.^39^ This validated and reliable tool is well-used in cancer survivors undergoing *and* beyond treatment,^40^ and it captures key domains of HRQoL/key symptoms relevant to the study population.	✓	✓	✓	
*Secondary outcomes*						
Patient	Effectiveness					
	Patient experience of care	Self-report: Patient Assessment of Care for Chronic Conditions includes 20 items derived from the '5As' model (ask, advise, agree, assist and arrange), a patient-centred model of behavioural counselling that is congruent with the Chronic Care Model and has been frequently used to enhance self-management support and linkages to community resources.		✓	✓	
	Symptom burden and distress	Self-report: Memorial Symptom Assessment Scale (MSAS)^48^ including MSAS-GDI (Global Distress Index), MSAS-PHYS (physical symptom distress) and MSAS-PSYCH (psychological symptom distress) subscales. This tool is valid and reliable in measuring symptom prevalence, characteristics, frequency, severity and distress in patients with cancer.^48^	✓	✓	✓	
	Cancer-related fatigue	Self-report: Brief Fatigue Inventory. This tool is reliable in assessing the severity of fatigue and the impact of fatigue on daily functioning in patients with cancer.	✓	✓	✓	
	Comorbidity burden	Self-report: A suite of questions from the Self-Administered Comorbidity Questionnaire,^49^ Charleston Comorbidity Index and Medicare Comorbidity list.	✓		✓	
	Diet and physical activity behaviour	Self-report: Short dietary questions related to fruit and veg intake^50^; Active Australia Survey^51^ and single item from the International Physical Activity Questionnaire short-form.^52^	✓	✓	✓	
	Financial Toxicity (FT)	Self-reports: 11-item COmprehensive Score for financial Toxicity-Functional Assessment of Chronic Illness Therapy tool.^53^ This tool is valid and reliable in measuring FT in patients with cancer.^54^	✓	✓	✓	
	Satisfaction of care	0–10 numerical analogue scale (with 10 being the most satisfied) supplemented with short qualitative interviews.			✓	✓
	Unplanned hospital admissions	Self-report (verified with hospital records)		✓	✓	✓
	Adherence to annual mammogram, physical breast exam and endocrine therapy	Hospital records			✓	✓
Cancer Centre	Reach and representativeness					
	Demographics	Self-report: age, gender, ethnicity, education, living arrangements, marital status, children (including ages) and employment	✓			
	Medical history	Hospital records and self-report: Previous cancer diagnoses and treatments	✓			
	Clinical characteristics	Hospital records and self-report: Breast cancer diagnosis and treatment protocols received	✓			
	Adoption and maintenance (sustainability)					
	% GPs explicit engagement in case conference	Records in database				✓
	Implementation					
	Completion (Y/N) and duration (min) of BCN-led consultation	BCN records, completion of the BCN-led consultation checklist and RN records in database				✓
	Completion (Y/N) and duration (min) of Case Conferencing with GP	BCN records, completion of the GP Case-conference checklist.				✓
	Completed survivorship care plan/components (Y/N)	BCN records, Survivorship Care Plans				✓
	Completed Chronic Disease Management plan (Y/N)	MBS (by GP)				✓
	No. and timing of GP visits and specialist visits	MBS and patient self-report (verified with hospital records)				✓
	Health provider satisfaction	Semistructured interviews				✓
	Economic appraisal					
	Cost-effectiveness	Self-report: The EQ-5D-5L is a standardised measure of health status developed by the EuroQol Group to provide a simple, generic measure of health for clinical and economic appraisal.^55^ The EQ-5D 5 L is a valid, reliable and responsive instrument with published Australian value sets for calculating quality-adjusted life years	✓	✓	✓	

T1=baseline/enrolment; T2=6 months post enrolment; T3=12 months post enrolment.

BCN, Breast Cancer Nurse; CDM, Chronic Disease Management plan; EQ 5D-5L, EuroQol 5 Dimensions 5 Levels; GP, primary care physician; MBS, Medicare Benefits Scheme; RE-AIM, Reach, Effectiveness, Adoption, Implementation and Maintenance.

### Implementation outcomes

The implementation of *IBIS-Survivorship* will be assessed according to the five components of the RE-AIM Framework,^25^ with outcomes and data collection methods summarised in table 2. Relevant outcomes include representativeness of the patient population according to demographic and clinical characteristics (*reach*), proportion of GPs that explicitly engage in the intervention (*adoption*), attendance rates and process outcomes of the BCN consultation and GP case conference such as how long it took to run each session and the percentage of planned components within each that were implemented (*implementation*) and key factors that facilitate or hinder the sustainability of the *IBIS-Survivorship* model (*maintenance*).

### Cost-effectiveness

A cost-effectiveness analysis will be undertaken to assess whether the *IBIS-Survivorship* model provides value for money compared with usual care. Additionally, costs of the intervention and health service use will be aggregated and patient benefits assessed. Intervention costs will be monitored by the study coordinator. Health service use will be captured on linked national administrative health datasets. Medicare Benefits Scheme and PBS data will be obtained from participants who provide consent to access this data and will include relevant claims details (ie, date of service, Medicare item number and description), cost details (ie, provider charge, scheduled fee, benefit paid, out of pocket expenses and billing type), service provider and referrer details (ie, provider specialty and item category). Unique study codes will be assigned to participants to link records uniquely and anonymously for analysis and ensure confidentiality is maintained. Strict data typographical verification protocols and consistency check protocols will be employed. Similarities and differences in datasets will be identified before results are aggregated.

### Data collection

#### Patient-reported data

Participant survey data will be collected and managed using REDCap electronic data capture tools hosted at Queensland University of Technology^27 28^ at three time periods (T1 – T3). All participants (intervention and control) will have the option to be emailed a link to online surveys or sent paper-based surveys. If surveys are not completed within 2 weeks of sending, a research nurse will contact the patient to encourage them to complete it.

#### Clinical data

Information about the participant’s cancer (histology, grade and stage of disease at diagnosis), date of diagnosis and treatment (surgery, chemotherapy and radiotherapy doses and dates) is obtained from medical records at T1 and entered into the REDCap database. Information will be obtained from health service medical records relating to hospital admissions and follow-up appointments including physical breast exam, mammography and endocrine therapy adherence occurring within the first 12 months post enrolment.

#### Stakeholder interviews

When they initially consent to the trial, participants will be invited to indicate agreement to participate in an optional interview to explore their experience of the *IBIS-Survivorship* shared care model. Patients allocated to the *IBIS-Survivorship* intervention who indicated a willingness to be approached for the interview will be invited to participate between 13 months and 24 months post enrolment. Participants will be informed that if they do not wish to participate in the interview, their current and future medical care will not be affected. In total, approximately 30 participants will be selected for the qualitative interviews based on maximum variation sampling to capture a broad range of perspectives. Additionally, all health professionals who contribute to the implementation of the *IBIS-Survivorship* model (BCNs, oncology specialists and GPs) will be invited to participate in an interview after completion of the trial to describe their satisfaction with the model, as well as barriers and facilitators experienced when implementing the intervention. Health professionals will be free to opt out of the interview process, and verbal acceptance to take part in an interview will be considered implied consent.

All interviews will be conducted via telephone or videoconferencing by a researcher skilled in qualitative interview techniques using the interview guide provided in online supplemental material BC. Participants will be encouraged to speak openly and to describe their experience with the *IBIS-Survivorship* intervention. All interviews will be audiorecorded and transcribed verbatim for analysis. It is anticipated that interviews will take approximately 30 min per participant.

### Sample size

The sample size is fixed by the number of clusters, the study duration and funding constraints. Initially, it was expected that seven cancer centres (clusters) would participate in the study; however, one cancer centre was unable to participate due to COVID-19 impacts on the health service, thus the sample size calculation and randomisation were determined after the number of clusters was confirmed as six. It is anticipated that the total number of available patients with early breast cancer will be 1384; split across the six clusters and nine cluster periods in the design. Assuming 22% of patients cannot identify a regular GP or decline participation (based on Cancer Australia’s pilot study),^29^ the potential sample size for recruitment is 1079. We will also allow for attrition of 30% by 12 months or T3 (conservative estimation based on the PROCARE Trial),^16^ thus the expected sample size for patient-reported data collection at T3 is 755 (equivalent to an average of 13 per cluster period and 125 per cluster, although varying cluster sizes are expected).

Using the online RShiny App,^41^ we estimated the power to detect a minimum clinically important difference in primary outcome (FACT-B mean scores at 12 months) between groups, of 8 points and a SD of 19 (total score possible=148), with a 2-sided type I error rate of 0.05. There is little information to support likely values of the intracluster correlation coefficient (ICC), the cluster autocorrelation coefficient (CAC) and the coefficient of variance (CV), so values were estimated from the literature. We allowed for clustering (assuming a within-period ICC of 0.03 and CV of 0.3) and for varying cluster sizes.^35^ To explore sensitivity, ICC estimates of 0.03 (0.005–0.05)^25^ and CV estimates of 0.3 (0.1–0.5) were used. Due to the longitudinal nature of this trial, correlations may differ in the same cluster period and in different cluster periods. Adjustment for time-varying correlations is made using a discrete time decay correlation structure, and the CAC is used to adjust for the correlation between two means in the same cluster at different times. Sensitivity of the CAC was explored using scores of 0.8 (0.6–0.9) in power calculations.^26^ Under these assumptions, the power curves reveal that under most anticipated scenarios, the trial will have 90% power in some conservative but plausible scenarios.

### Randomisation and blinding

The unit of randomisation is the cluster. Using the SW-CRT design, each cluster will begin the study as a control site, providing usual care follow-up to participants as per figure 1. An independent statistician randomly generated the order of the clusters, which will be concealed from all investigators and revealed to the cluster investigators 8 weeks prior to the crossover date by the project coordinator. All other clusters will be blinded to the site randomisation. Covariate constrained randomisation was used to balance the number of cluster-periods from a metro and regional cluster across the intervention and control periods. All possible randomisation schemes (720) were simulated. The balance of the randomisation schemes was then scored using the weighted squared difference in the mean values of regional across the intervention and control cluster-periods (where the weight was the inverse variance of the cluster means). A randomisation scheme was selected from the 20% of schemes with the best balance.

During each cluster period, research nurses at each cluster will identify eligible breast cancer patients and invite them to participate in the study. The research nurse recruiting participants will be blind to the randomisation sequence. The randomisation schedule will be kept centrally and only accessible by the project coordinator. The BCNs at each cluster will be informed 8 weeks prior to the crossover date when they will implement the intervention, to allow time for training in intervention delivery. The BCNs will only deliver the intervention to participants enrolled after the cluster’s crossover date. Participants are unable to be blinded and will be informed by their BCN whether they are receiving usual care (control) or *IBIS-Survivorship* care (intervention) at their first clinical appointment following enrolment to the study.

### Data analysis

Provided below is an overview of the statistical analysis plan. Analyses will be conducted independently by statisticians at Queensland University of Technology, using STATA V.18.

Following data cleaning, frequencies will be generated for all variables. Scales will be created, and descriptive statistics determined according to the distribution. Differences in demographic and medical characteristics between intervention and control groups at baseline will be described using numbers and percentages, means and SD or medians and IQRs as appropriate. Every effort will be made to follow up with participants to minimise any missing data as a potential for bias. Missing data patterns will be investigated, but there will be no imputation for missing data and analyses will only be conducted on outcome variables with complete data. The following analyses will be conducted under the assumption of missing at random.

#### Analysis of effectiveness outcomes

Analyses will be conducted as intention to treat, and clusters will be considered exposed to the intervention postrandomised crossover date. This will include all eligible patients for whom data have been obtained, from enrolment to 12 months. Data will be analysed with respect to the treatment specified for each cluster by the allocated randomisation order.

The primary and secondary continuous outcomes will be analysed using a linear mixed model with an identity link function using a restricted maximum likelihood approximation with a Kenward-Roger approximation which is suitable for small samples.^42 43^ Results will be presented as mean differences between groups with 95% CIs.

The primary outcome will be assessed in a model which will include a random cluster effect, a fixed effect for period (1-9) and a random cluster by period effect to test the difference in means between groups (usual care vs intervention) for FACT-B scores at 12 months. This will estimate the time-averaged effect of the intervention. The random cluster by period interactions will allow for correlations within clusters to decay with increasing separation between times of measurement of observations. Adjustments will be made for the baseline FACT-B scores (as a covariate) and for factors used in the randomisation procedure (metropolitan vs regional location of cancer centre).

Sensitivity analyses will be conducted to estimate the effect of time since exposure, where time since exposure is a fixed effect (1-8). This will assess the effect of the intervention after one period of exposure, two periods of exposure and up to eight periods of exposure compared graphically in forest plots. Additional sensitivity analyses will adjust for patient level covariates (eg, age, education, living arrangement, disease or treatment variable and number of comorbidities) based on expert clinical guidance. The selection of baseline characteristics is based on clinical concern for imbalance between intervention groups or variables that are most prognostic of the outcome.

Similar analyses will be conducted for all secondary outcomes at 6 months and 12 months, including analysis of the difference in means between groups for FACT-B scores at 6 months. Results will be presented as mean differences between groups with 95% CIs.

A per protocol analysis will be completed for primary and secondary outcomes of the trial, which will exclude departures from randomisations such as participants randomised to receive the intervention who did not receive the intervention. These participants will be removed from the analysis. The two key components of the intervention (nurse-led consultation and GP case conference) will be considered intervention ‘doses’ and the per protocol analysis will be used to compare groups who completed either the full intervention or half the intervention with those on usual care. A further analysis will use the actual cluster cross-over date if there is variation from the proposed crossover date.

#### Analysis of implementation outcomes

To understand key factors that facilitate or hinder the implementation of the *IBIS-Survivorship* quantitative process, measures collected in alignment with the RE-AIM indicators will be summarised and reported using descriptive statistics. Analysis of the qualitative interviews will be guided by the Consolidated Framework for Implementation Research using both inductive and deductive methods. Inductive analysis will use reflexive thematic analysis as described by Braun and Clarke. Coding will be performed with the use of the NVivo V.12 software programme.

#### Cost-effectiveness analysis

For the cost-effectiveness analysis, we will compare the costs and patient well-being associated with the intervention versus usual care, by incorporating a societal cost perspective. We will assess cost-effectiveness and model longer-term predicted outcomes based on real-world evidence synthesis. We will also assess BCN costs to carry out and deliver the intervention (time and salary). The economic evaluation, modelling and budget impact analysis^44^ will conform to international best practice methods.^44^,^46^ We will consider intermediate outcomes of *IBIS-Survivorship* linked to patient well-being (eg, % GP adoption influencing cost-savings, symptom improvement leading to earlier return to work). Incremental cost per effect ratios will be generated which represent the additional cost and quality-adjusted life years of the intervention over and above that of usual care. One-way and probabilistic sensitivity analyses will be performed to address uncertainty and scenario analyses. Data will be analysed in TreeAge Pro for Healthcare 2024 software.

#### Interim analyses

No interim analyses are planned.

### Trial and data management

All data will be recorded in electronic case report forms directly entered into REDCap.^27 28^ All data entered will be recorded under a participant code (re-identifiable) to maintain confidentiality. Research staff will receive training regarding data collection and data entry from the project coordinator. All data are stored on a secure server with questionnaire data kept separate from personal information, which is only accessible to key project staff.

To maximise data integrity and completeness, the project coordinator will undertake weekly audits with data validation. The project coordinator will oversee the data monitoring of the trial and monitor the safety of patients enrolled in the trial, independently from principal and site investigators.

All original signed consent forms will be retained in accordance with institutional policy, and a copy of the signed PICF will be provided to the participant. The date that informed consent was given will be recorded on the REDCap case report forms.

### Adverse events

There are no known side effects or adverse events associated with the proposed intervention. Due to the nature of the intervention, there will be no reporting of adverse events related to the intervention. There is a very small possibility that participants might experience distress as they answer questions in the case report form in relation to their symptoms, disease or experience of care. While the research team is not expecting any distress caused specifically by this research, if any participant experiences distress as a result of this trial, we will refer them to their relevant clinical teams for appropriate actions. We will also provide the Cancer Council Information and Support Line (phone 13 11 20) which is a free, confidential telephone service run by Cancer Councils in each state and territory in Australia.

### Participant withdrawal

Participants are free to withdraw from the study without giving a reason, at any time. If a participant decides to withdraw, all efforts will be made to complete and report the observations, especially the listed primary and secondary objectives, as thoroughly as possible up to the date of withdrawal. The primary reason for withdrawal (where known) will be identified and recorded, along with the date of withdrawal.

### Patient and public involvement

The current trial was developed based on a pilot trial conducted at a single site.^15 47^ Following completion of the pilot trial, cancer survivors and health providers were invited to have input into the current study design. This input process was supported by the Primary Care Collaborative Cancer Clinical Trials Group (PC4). Additionally, breast cancer survivors were invited to provide comments on the current study protocol and ethical issues. The intervention model of care has been developed in close partnership with the McGrath Foundation, which is a charity organisation in Australia that works closely with breast cancer survivors and BCNs.

### Ethics and dissemination

The study will be conducted in full conformance with principles of the ‘Declaration of Helsinki’ (recommendations guiding medical doctors in biomedical research involving human subjects), Notes for Guidance on Good Clinical Practice (CPMP/ICH/135/95) and with the National Health and Medical Research Council National Statement on Ethical Conduct in Research Involving Humans (2007 and 2019). This study has received National Mutual Acceptance approval from the Metro South Human Research Ethics Committee (HREC/2020/QMS/59892) with Site Specific Assessment Approval obtained from all participating sites.

It is intended that the findings from this trial will be disseminated at academic and professional scientific meetings and publications in peer-reviewed journals. Authorship will be based on the International Committee of Medical Journal Editors guidelines. Participants will be identified in such reports only in aggregate. Trial results will also be disseminated to all research participants with a summary sheet that will outline the relevant findings in lay language.

### Protocol amendments and deviation

Neither the principal investigator nor the recruitment sites will modify or alter this protocol without the agreement of all others. All agreed protocol amendments will be clearly recorded on a protocol amendment form and will be signed and dated by the original protocol approving signatories. All protocol amendments will be submitted to the Metro South Health Human Research Ethics Committee (MSH HREC) for approval before implementation. The only exception will be when the amendment is necessary to eliminate an immediate hazard to the trial participants. In this case, the necessary action will be taken first, with the relevant protocol amendment following shortly thereafter. Should any protocol deviation occur, it will be reported to the study project manager as soon as is practical. The deviation and the reason for its occurrence will be included in the study report.

## Supplementary material

10.1136/bmjopen-2025-103341 online supplemental file 1

## Data Availability

Data sharing not applicable as no datasets generated and/or analysed for this study.
