# Supplementary material for: Implementation and effectiveness of a nurse-enabled, shared-care follow-up model for early breast cancer survivors (The IBIS-Survivorship Study): protocol for a stepped-wedge cluster randomised trial
Source: BMJ Open. 2025 Jun 19;15(6):e103341. doi: 10.1136/bmjopen-2025-103341 (PMC12182111; doi:10.1136/bmjopen-2025-103341)
Supplement: online supplemental file 1 [file bmjopen-15-6-s001.docx]

**Supplementary Material A**

# Reporting checklist for protocol of a clinical trial.

Based on the SPIRIT guidelines.

| **Administrative information** |  | **Reporting Item** | **Page Number** |
| --- | --- | --- | --- |
|  |  |  |  |
| Title | [#1](https://www.goodreports.org/reporting-checklists/spirit/info/#1) | Descriptive title identifying the study design, population, interventions, and, if applicable, trial acronym | 1 |
| Trial registration | [#2a](https://www.goodreports.org/reporting-checklists/spirit/info/#2a) | Trial identifier and registry name. If not yet registered, name of intended registry | 1 |
| Trial registration: data set | [#2b](https://www.goodreports.org/reporting-checklists/spirit/info/#2b) | All items from the World Health Organization Trial Registration Data Set |  |
| Protocol version | [#3](https://www.goodreports.org/reporting-checklists/spirit/info/#3) | Date and version identifier | 3 |
| Funding | [#4](https://www.goodreports.org/reporting-checklists/spirit/info/#4) | Sources and types of financial, material, and other support | 18 |
| Roles and responsibilities: contributorship | [#5a](https://www.goodreports.org/reporting-checklists/spirit/info/#5a) | Names, affiliations, and roles of protocol contributors | Title page |
| Roles and responsibilities: sponsor contact information | [#5b](https://www.goodreports.org/reporting-checklists/spirit/info/#5b) | Name and contact information for the trial sponsor | 18 |
| Roles and responsibilities: sponsor and funder | [#5c](https://www.goodreports.org/reporting-checklists/spirit/info/#5c) | Role of study sponsor and funders, if any, in study design; collection, management, analysis, and interpretation of data; writing of the report; and the decision to submit the report for publication, including whether they will have ultimate authority over any of these activities | 18 |
| Roles and responsibilities: committees | [#5d](https://www.goodreports.org/reporting-checklists/spirit/info/#5d) | Composition, roles, and responsibilities of the coordinating centre, steering committee, endpoint adjudication committee, data management team, and other individuals or groups overseeing the trial, if applicable (see Item 21a for data monitoring committee) | 18 |
| **Introduction** |  |  |  |
| Background and rationale | [#6a](https://www.goodreports.org/reporting-checklists/spirit/info/#6a) | Description of research question and justification for undertaking the trial, including summary of relevant studies (published and unpublished) examining benefits and harms for each intervention | 2-3 |
| Background and rationale: choice of comparators | [#6b](https://www.goodreports.org/reporting-checklists/spirit/info/#6b) | Explanation for choice of comparators | 5 |
| Objectives | [#7](https://www.goodreports.org/reporting-checklists/spirit/info/#7) | Specific objectives or hypotheses | 3 |
| Trial design | [#8](https://www.goodreports.org/reporting-checklists/spirit/info/#8) | Description of trial design including type of trial (eg, parallel group, crossover, factorial, single group), allocation ratio, and framework (eg, superiority, equivalence, non-inferiority, exploratory) | 3 |
| **Methods: Participants, interventions, and outcomes** |  |  |  |
| Study setting | [#9](https://www.goodreports.org/reporting-checklists/spirit/info/#9) | Description of study settings (eg, community clinic, academic hospital) and list of countries where data will be collected. Reference to where list of study sites can be obtained | 3-4 |
| Eligibility criteria | [#10](https://www.goodreports.org/reporting-checklists/spirit/info/#10) | Inclusion and exclusion criteria for participants. If applicable, eligibility criteria for study centres and individuals who will perform the interventions (eg, surgeons, psychotherapists) | 3 |
| Interventions: description | [#11a](https://www.goodreports.org/reporting-checklists/spirit/info/#11a) | Interventions for each group with sufficient detail to allow replication, including how and when they will be administered | 4-5 |
| Interventions: modifications | [#11b](https://www.goodreports.org/reporting-checklists/spirit/info/#11b) | Criteria for discontinuing or modifying allocated interventions for a given trial participant (eg, drug dose change in response to harms, participant request, or improving / worsening disease) | 5 |
| Interventions: adherence | [#11c](https://www.goodreports.org/reporting-checklists/spirit/info/#11c) | Strategies to improve adherence to intervention protocols, and any procedures for monitoring adherence (eg, drug tablet return; laboratory tests) | 5 |
| Interventions: concomitant care | [#11d](https://www.goodreports.org/reporting-checklists/spirit/info/#11d) | Relevant concomitant care and interventions that are permitted or prohibited during the trial | 5 |
| Outcomes | [#12](https://www.goodreports.org/reporting-checklists/spirit/info/#12) | Primary, secondary, and other outcomes, including the specific measurement variable (eg, systolic blood pressure), analysis metric (eg, change from baseline, final value, time to event), method of aggregation (eg, median, proportion), and time point for each outcome. Explanation of the clinical relevance of chosen efficacy and harm outcomes is strongly recommended | 6 and Table 2 |
| Participant timeline | [#13](https://www.goodreports.org/reporting-checklists/spirit/info/#13) | Time schedule of enrolment, interventions (including any run-ins and washouts), assessments, and visits for participants. A schematic diagram is highly recommended (see Figure) | 6, Figure 1, Table 2 |
| Sample size | [#14](https://www.goodreports.org/reporting-checklists/spirit/info/#14) | Estimated number of participants needed to achieve study objectives and how it was determined, including clinical and statistical assumptions supporting any sample size calculations | 7 |
| Recruitment | [#15](https://www.goodreports.org/reporting-checklists/spirit/info/#15) | Strategies for achieving adequate participant enrolment to reach target sample size | 4 |
| **Methods: Assignment of interventions (for controlled trials)** |  |  |  |
| Allocation: sequence generation | [#16a](https://www.goodreports.org/reporting-checklists/spirit/info/#16a) | Method of generating the allocation sequence (eg, computer-generated random numbers), and list of any factors for stratification. To reduce predictability of a random sequence, details of any planned restriction (eg, blocking) should be provided in a separate document that is unavailable to those who enrol participants or assign interventions | 8 |
| Allocation concealment mechanism | [#16b](https://www.goodreports.org/reporting-checklists/spirit/info/#16b) | Mechanism of implementing the allocation sequence (eg, central telephone; sequentially numbered, opaque, sealed envelopes), describing any steps to conceal the sequence until interventions are assigned | 8 |
| Allocation: implementation | [#16c](https://www.goodreports.org/reporting-checklists/spirit/info/#16c) | Who will generate the allocation sequence, who will enrol participants, and who will assign participants to interventions | 8 |
| Blinding (masking) | [#17a](https://www.goodreports.org/reporting-checklists/spirit/info/#17a) | Who will be blinded after assignment to interventions (eg, trial participants, care providers, outcome assessors, data analysts), and how | 8 |
| Blinding (masking): emergency unblinding | [#17b](https://www.goodreports.org/reporting-checklists/spirit/info/#17b) | If blinded, circumstances under which unblinding is permissible, and procedure for revealing a participant’s allocated intervention during the trial | 8 |
| **Methods: Data collection, management, and analysis** |  |  |  |
| Data collection plan | [#18a](https://www.goodreports.org/reporting-checklists/spirit/info/#18a) | Plans for assessment and collection of outcome, baseline, and other trial data, including any related processes to promote data quality (eg, duplicate measurements, training of assessors) and a description of study instruments (eg, questionnaires, laboratory tests) along with their reliability and validity, if known. Reference to where data collection forms can be found, if not in the protocol | 6-7 |
| Data collection plan: retention | [#18b](https://www.goodreports.org/reporting-checklists/spirit/info/#18b) | Plans to promote participant retention and complete follow-up, including list of any outcome data to be collected for participants who discontinue or deviate from intervention protocols | 7 |
| Data management | [#19](https://www.goodreports.org/reporting-checklists/spirit/info/#19) | Plans for data entry, coding, security, and storage, including any related processes to promote data quality (eg, double data entry; range checks for data values). Reference to where details of data management procedures can be found, if not in the protocol | 10 |
| Statistics: outcomes | [#20a](https://www.goodreports.org/reporting-checklists/spirit/info/#20a) | Statistical methods for analysing primary and secondary outcomes. Reference to where other details of the statistical analysis plan can be found, if not in the protocol | 8-9 |
| Statistics: additional analyses | [#20b](https://www.goodreports.org/reporting-checklists/spirit/info/#20b) | Methods for any additional analyses (eg, subgroup and adjusted analyses) | 9 |
| Statistics: analysis population and missing data | [#20c](https://www.goodreports.org/reporting-checklists/spirit/info/#20c) | Definition of analysis population relating to protocol non-adherence (eg, as randomised analysis), and any statistical methods to handle missing data (eg, multiple imputation) | 8-9 |
| **Methods: Monitoring** |  |  |  |
| Data monitoring: formal committee | [#21a](https://www.goodreports.org/reporting-checklists/spirit/info/#21a) | Composition of data monitoring committee (DMC); summary of its role and reporting structure; statement of whether it is independent from the sponsor and competing interests; and reference to where further details about its charter can be found, if not in the protocol. Alternatively, an explanation of why a DMC is not needed | 10 |
| Data monitoring: interim analysis | [#21b](https://www.goodreports.org/reporting-checklists/spirit/info/#21b) | Description of any interim analyses and stopping guidelines, including who will have access to these interim results and make the final decision to terminate the trial | 10 |
| Harms | [#22](https://www.goodreports.org/reporting-checklists/spirit/info/#22) | Plans for collecting, assessing, reporting, and managing solicited and spontaneously reported adverse events and other unintended effects of trial interventions or trial conduct | 10 |
| Auditing | [#23](https://www.goodreports.org/reporting-checklists/spirit/info/#23) | Frequency and procedures for auditing trial conduct, if any, and whether the process will be independent from investigators and the sponsor | 10 |
| **Ethics and dissemination** |  |  |  |
| Research ethics approval | [#24](https://www.goodreports.org/reporting-checklists/spirit/info/#24) | Plans for seeking research ethics committee / institutional review board (REC / IRB) approval | 11 |
| Protocol amendments | [#25](https://www.goodreports.org/reporting-checklists/spirit/info/#25) | Plans for communicating important protocol modifications (eg, changes to eligibility criteria, outcomes, analyses) to relevant parties (eg, investigators, REC / IRBs, trial participants, trial registries, journals, regulators) | 11 |
| Consent or assent | [#26a](https://www.goodreports.org/reporting-checklists/spirit/info/#26a) | Who will obtain informed consent or assent from potential trial participants or authorised surrogates, and how (see Item 32) | 4 |
| Consent or assent: ancillary studies | [#26b](https://www.goodreports.org/reporting-checklists/spirit/info/#26b) | Additional consent provisions for collection and use of participant data and biological specimens in ancillary studies, if applicable | 4 |
| Confidentiality | [#27](https://www.goodreports.org/reporting-checklists/spirit/info/#27) | How personal information about potential and enrolled participants will be collected, shared, and maintained in order to protect confidentiality before, during, and after the trial | 10 |
| Declaration of interests | [#28](https://www.goodreports.org/reporting-checklists/spirit/info/#28) | Financial and other competing interests for principal investigators for the overall trial and each study site | 18 |
| Data access | [#29](https://www.goodreports.org/reporting-checklists/spirit/info/#29) | Statement of who will have access to the final trial dataset, and disclosure of contractual agreements that limit such access for investigators | 10 |
| Ancillary and post trial care | [#30](https://www.goodreports.org/reporting-checklists/spirit/info/#30) | Provisions, if any, for ancillary and post-trial care, and for compensation to those who suffer harm from trial participation | 10 |
| Dissemination policy: trial results | [#31a](https://www.goodreports.org/reporting-checklists/spirit/info/#31a) | Plans for investigators and sponsor to communicate trial results to participants, healthcare professionals, the public, and other relevant groups (eg, via publication, reporting in results databases, or other data sharing arrangements), including any publication restrictions | 11 |
| Dissemination policy: authorship | [#31b](https://www.goodreports.org/reporting-checklists/spirit/info/#31b) | Authorship eligibility guidelines and any intended use of professional writers | 11 |
| Dissemination policy: reproducible research | [#31c](https://www.goodreports.org/reporting-checklists/spirit/info/#31c) | Plans, if any, for granting public access to the full protocol, participant-level dataset, and statistical code | 11 |
| **Appendices** |  |  |  |
| Informed consent materials | [#32](https://www.goodreports.org/reporting-checklists/spirit/info/#32) | Model consent form and other related documentation given to participants and authorised surrogates | Supplementary Materials |
| Biological specimens | [#33](https://www.goodreports.org/reporting-checklists/spirit/info/#33) | Plans for collection, laboratory evaluation, and storage of biological specimens for genetic or molecular analysis in the current trial and for future use in ancillary studies, if applicable | N/A |

**Supplementary Material B**

| **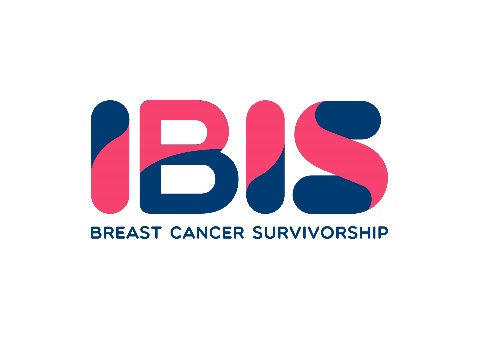** | **[INSERT INSTITUTION LOGO]** |
| --- | --- |

**MASTER Participant Information Sheet/Consent Form for Patients**

| **Title** | Implementation of a nurse-enabled, shared-care follow-up model for early breast cancer survivors |
| --- | --- |
| **Short Title** | The IBIS-Survivorship Study |
| **Coordinating Principal Investigator** | Professor Raymond Chan |
| **Principal Investigator** | *[Institution Principal Investigator]* |

# Would you like to take part in this trial?

You are invited to take part in this research project. This is because **you have or** **will be** **completing treatment for early breast cancer soon**. The research project is testing a new model of care for breast cancer survivorship. The new model of care is called *IBIS-Survivorship*.

This Participant Information Sheet/Consent Form tells you about the research project. It explains the tests and treatments involved. Knowing what is involved will help you decide if you want to take part in the research. Please read this information carefully. Ask questions about anything that you don’t understand or want to know more about. Before deciding whether or not to take part, you might want to talk about it with a relative, friend or your local doctor.

# What is the purpose of this research?

In this trial, we test a nurse-coordinated model of follow-up care, called *IBIS-Survivorship*, where the responsibilities for your care are shared between your cancer specialists (a surgeon, an oncologist, and an oncology nurse) and your general practitioner (GP). This model of care is coordinated by a specialist nurse and is designed to find out if *IBIS-Survivorship* can improve your quality of life and experience with health care during your post-treatment follow up.

The current models of care often require patients to frequently attend the cancer centre. The follow-up appointment schedules and the involvement of each healthcare practitioner (including the GP) can vary from patient-to-patient, depending on the facility where they are being seen and as decided by each healthcare practitioner.

We propose that a standardised, coordinated model of follow-up care involving your cancer specialists and GP would be beneficial for your health and experience of care.

This research has been initiated by the study doctor, Professor Raymond Chan. This research has been funded by the National Health and Medical Research Council (NHMRC) Partnership Grant.

# Your participation is voluntary

Your participation in this study is completely voluntary and there will be no cost to you. If you do not want to take part in this study you do not have to. You should feel under no obligation to participate in this study. Choosing not to take part in this study will not affect your current and future medical care in any way

# Your withdrawal from the study

You are under no obligation to continue with the research study. You may change your mind at any time about participating in the research. People withdraw from studies for various reasons and you do not need to provide a reason.

You can withdraw from the study at any time by completing and signing the ‘**Participant Withdrawal of Consent Form**’. This form is provided at the end of this document, and is to be completed by you and supplied to the research team if you choose to withdraw at a later date.

If you withdraw from the study, you will be able to choose whether the study will destroy or retain the information it has collected about you. You should only choose one of these options. Where both boxes are ticked in error or neither box is ticked, the study will destroy all information it has collected about you.

# What does participation in this research involve?

If you decide you want to take part in the research project, you will be asked to sign the consent section. By signing it you are telling us that you:

- Understand what you have read
- Consent to take part in the research project
- Consent to have the tests and treatments that are described
- Consent to the use of your personal and health information as described
- You will be given a copy of this Participant Information and Consent Form to keep.

## What do I need to do?

If you agree to participate, a research nurse will provide you with detailed information about the study. You will be participating in a stepped wedge trial. Sometimes we do not know which model of care is best for following-up patients after treatment. To find out we need to compare the different models of care. In this stepped wedge trial, each study hospital will start by offering only usual care to new participants. Following this, at regular intervals, or “steps”, a study hospital will be randomly allocated to only offer *IBIS-Survivorship* to new participants. During the trial, each participant will only receive the model of care that they are assigned to when they start the trial.

This research project has been designed to make sure the researchers interpret the results in a fair and appropriate way and avoids study doctors or participants jumping to conclusions. There are no additional costs associated with participating in this research project, nor will you be paid. All medication, tests and medical care required as part of the research project will be provided to you free of charge. If you decide to participate in this research project, the study doctor will inform your local doctor.

**If you are enrolled in the study while** *[Institution Name]* **is offering *IBIS-Survivorship* model of follow-up care,** you will have a range of appointments offered by your care team including oncologists, surgeons, cancer nurses and your GP. These appointments take place over a period of 5 years after your treatment completion and each will have a different focus and may be face-to-face or by video/teleconferencing.

1. ***Specialist nurse appointment (30-60 minutes)***: 6-10 weeks after you have finished treatment, you will be scheduled to see your specialist cancer nurse. During this appointment, your nurse will provide you with:

- a treatment summary
- Education about post-treatment care, symptom management, healthy lifestyle
- a Survivorship Care Plan, including:
  - follow-up appointment schedule tailored to your needs and any treatment you had
  - list of the responsibilities of all healthcare providers involved (including your hospital cancer care team and your GP)

1. Your specialist nurse will communicate all care you received in the cancer centre including the treatment summary and Survivorship Care Plan with your GP, and seek his/her input. Your appointment with the specialist nurse and their communication with the GP will be audio-/video-recorded for further analysis to help the researcher understand the useful processes and components within our care.
2. **Initial GP appointment (duration as per GP):** You will be encouraged to see your GP at around 8 weeks, so that he/she can discuss the care plan with you also from his/her perspective.
3. **GP and Cancer Specialist appointments (duration as per specialist or GP):** You will be provided with a schedule of 5 appointments for your GP and 6 appointments for your Cancer Specialists for up to five years after diagnosis. You are not required to memorise all appointments as these will be listed in the Survivorship Care Plan, and this plan will be provided to you and your healthcare professionals. Also, you will be reminded before every follow-up appointment.

**If you are enrolled in the study while** *[Institution Name]* **is still offering their *Usual* model of follow-up care,** you will be cared for by your surgeon, oncologists, nurses and other healthcare professionals as usual. There will be no change to your care except, we will provide you with an additional information booklet on “Living Well After Cancer” published by Cancer Council Australia.

## Complete Questionnaires (20-30 minutes)

Whether you receive *IBIS-Survivorship* or Usual follow-up care, a research assistant will contact you at three points during the study period to ask you to complete questionnaires so that we can find out about your health/healthcare experience before you start the trial and at 6 months and 12 months after you commenced on the trial. You will be able to complete these questionnaires by online survey, over the telephone or by paper survey. The questionnaires will take about 20-30 minutes to complete (depending on how you choose to complete them) and a little longer at the first time point. The research staff may send reminders to you via phone call, text or email, as required.

**It should be noted that the questions will not be reviewed by a health professional and you will need to contact an appropriate practitioner (i.e. your specialist cancer nurse) if you have concerns about your health.** Your specialist cancer nurse will provide you with this number at the completion of your cancer treatment.

## Medicare Benefits Schedule (MBS) and Pharmaceutical Benefits Scheme (PBS) Consent Form

We also want to understand whether or not the models of care we are studying influence people’s need to use healthcare services and medications outside of hospital**. You will be asked to sign a consent form authorising the study to access to your complete Medicare Benefits Schedule (MBS) and Pharmaceutical Benefits Scheme (PBS) data** as outlined in the consent form. Medicare collects information on your doctor visits and the associated costs, while the PBS collects information on the prescription medications you have filled at pharmacies.

The consent form is sent securely to the Services Australia who holds MBS and PBS and data confidentially.

A sample of the types of coded claims information that will be provided is on the last page of the consent form. This claims information will help us to understand whether or not people used less (or more) healthcare appointments and medications after starting this study.

## Complete Interview (Optional, 30 minutes)

A research assistant will contact you 12 months after you commenced on the trial and ask you to participate in a one off, individual interview to find out about your experience of participating in the trial. This interview is voluntary and will be audio-recorded to allow the research team to reflect and analyse the interview data at a later date. The interview should take no longer than about 30 minutes.

# Other relevant information about the research project

The methods of this research study, including the use of all the questionnaires, have been tested in a small pilot study of 60 participants at a large metropolitan tertiary teaching hospital. The implementation of the *IBIS-Survivorship* model of follow-up care is now being tested in 7 study hospitals across Australia. We will be enrolling 1,358 participants across all the study hospitals. This study involves researchers working in collaboration from several institutions across Australia.

# What are the alternatives to participation?

You do not have to take part in this research project to receive treatment at this hospital. Other options are available. Whether or not you choose to participate in this research, you will still be offered the model of care that is being offered at *[Name of Institution]*. Your study doctor will discuss these options with you before you decide whether or not to take part in this research project. You can also discuss the options with your local doctor.

# What are the possible benefits of taking part?

We cannot guarantee or promise that you will receive any benefits from this research; however, possible benefits may include an improvement in your health and experience of care from the post-treatment follow up care approach (i.e. appointments and interventions we are trialling).

# What are the possible risks and disadvantages of taking part?

There are minimal risks associated with your participation in this project.

## Distress

There is a very small possibility that participants might experience distress as they answer questions in the questionnaires in relation to their symptoms, disease, or experience of care. If you become upset or distressed as a result of your participation in the research, the study doctor will be able to arrange for counselling or other appropriate support. Any counselling or support will be provided by qualified staff who are not members of the research project team. This counselling will be provided free of charge. Alternatively, Lifeline provides access to online, phone or face-to-face support, call **13 11 14** for 24-hour telephone crisis support.

# What if new information arises during this research project?

Sometimes during the course of a research project, new information becomes available about the treatment that is being studied. If this happens, your study doctor will tell you about it and discuss with you whether you want to continue in the research project. If you decide to withdraw, your study doctor will make arrangements for your regular health care to continue. If you decide to continue in the research project you will be asked to sign an updated consent form.

Also, on receiving new information, your study doctor might consider it to be in your best interests to withdraw you from the research project. If this happens, he/she will explain the reasons and arrange for your regular health care to continue.

# Your withdrawal from the study

If you decide to withdraw from the project, you will still be offered the model of care that is being offered at *[Name of Institution]*. You can choose to withdraw from

- the whole study: where we stop collecting any data about you **OR**
- part of the study involving your active participation (i.e., completing questionnaires and participating in the interview) **AND/OR**
- part of the study relating to your MBS and PBS claims.

You are under no obligation to continue with the research study. You may change your mind at any time about participating in the research. People withdraw from studies for various reasons and you do not need to provide a reason.

You can withdraw from the study at any time by completing and signing the ‘Participant Withdrawal of Consent Form’. This form is provided at the end of this document, and is to be completed by you and supplied to the research team if you choose to withdraw at a later date.

If you withdraw from the study, you will be able to choose whether the study will destroy or retain the information it has collected about you. You should only choose **one** of these options. Where both boxes are ticked in error or neither box is ticked, the study will destroy all information it has collected about you.

# What happens when the research project ends?

We will not contact you again after the 5 year follow-up period for this study. At 5 years, you will be discharged to the care of your GP, as is standard practice or followed-up according to your hospital’s policy.

# Storage, retention and destruction of your information

By signing the consent form, you consent to the study doctor and relevant research staff collecting and using personal information about you for the research project. The following will happen:

## General Study Data

- Information about you will be obtained from your health records held at this and other health services or collected directly from you in the form of research questionnaires and an interview (if relevant) and stored securely at *[Name of institution]* and on a secure server at Queensland University of Technology.
- Access to your health records will occur over a five-year period.
- Audio-/video-recordings of the nurse-led clinics and GP case conferences (and relevant checklists) will be recorded on a device and transferred from *[Name of institution]* to Queensland University of Technology via the Research Data Storage Service (RDSS) within 7 days of collection. This service ensures that data is encrypted during upload and download and is stored in Australian Data Centres. Once the integrity of the data transfer is confirmed by the research team, the audio-/video-recording will be deleted from the recording device.
- Information about your participation in this research project may be recorded in your health records.
- All information collected about you will be assigned a unique identification number and any identifying information will be removed from these data to protect your privacy. A ‘coding key’ that links your identifying information with your unique number will be stored separately in an electronic database only accessible by authorised personnel. It will be disclosed only with your permission, or as required by law.
- As this research involves linking types of data, the use of information that could be used to identify you may be required to ensure that the data is linked correctly. Once linking of the data types has been completed, any identifying information will be removed.
- Personal information about you, such as your name and address will NOT be used outside of this study. Any information obtained in connection with this research project that can identify you will remain confidential.
- Re-identifiable information that we collect about you, including medical records, will be kept for 15 years after the project is completed on a secure server (electronic data) or locked filing cabinet (physical data) at Queensland University of Technology.
- Those authorised persons responsible for analysing data will not have access to your identifying information (or the coding key).
- 15 years after the study is completed, your identifying information will be permanently deleted from the computer system and any hard copies will be securely destroyed. However, the re-identifiable/coded (it is possible to use the code to re-identify you) information held by the investigators will not be destroyed (see section 14.2, below, for the destruction of MBS/PBS data).
- A study report will be prepared and may be submitted to regulatory authorities and for publication and conference presentation. However, participants will be identified in such reports only by study identification number, gender and age.
- It is anticipated that the results of this research project will be published and/or presented in a variety of forums. In any publication and/or presentation, information will be provided in such a way that you cannot be identified, except with your permission. Results will be combined to be presented or published. If you would like to receive a copy of these published results, please indicate this on the consent form or contact Professor Raymond Chan and we will send you a copy with our compliments. Please note that individual results will not be available, results will only be provided in aggregate.
- Delegation logs about research activities including accessing health records for the purpose of data collection will be maintained at *[Name of institution]*.

## MBS/PBS Data

- Physical information (paper versions of documents, including consent forms) will be stored in a secure building with restricted access in a locked filing cabinet. Electronic information will be stored securely (encrypted) on a restricted access secure server connected to the internet using Queensland University of Technology RDSS.
- Access to your physical and/or electronic MBS/PBS data and consent forms (paper or electronic) will be restricted to members of the research team for the purposes of analysis and reporting. Access to this data will be maintained on a
- MBS/PBS data will not be used in any future, unspecified research outside of this study.
- All MBS/PBS data (stored, archived or held as back-ups) will be securely destroyed after 5 years from the publication of the project’s final report. Physical data will be destroyed using a secure waste bin and all electronic information will be securely destroyed as per QUT IT department policy.

Your health records and any information obtained during the research project are subject to inspection (for the purpose of verifying the procedures and the data) by the relevant authorities and authorised representatives of the Sponsor, Professor Raymond Chan, the institution relevant to this Participant Information Sheet, *[Name of institution]*, or as required by law. By signing the Consent Form, you authorise release of, or access to, this confidential information to the relevant study personnel and regulatory authorities as noted above.

**All data published will be combined and have identifying information removed**. The use of this data will comply with the requirements of the Australian Privacy Principles and the Privacy Act 1998 (Cth). In accordance with relevant Australian and *[Name of state/territory]* privacy and other relevant laws, **you have the right to request access to your information collected by the research team**. You also have the right to request that any information with which you disagree be corrected. Please contact the study team member named at the end of this document if you would like to access your information.

# Who is organising and funding the research?

This research project is being conducted by Professor Raymond Chan. Queensland University of Technology will receive a payment from National Health and Medical Research Council for undertaking this research project. No member of the research team will receive a personal financial benefit from your involvement in this research project (other than their ordinary wages).

# Who has reviewed the research project?

All research in Australia involving humans is reviewed by an independent group of people called a Human Research Ethics Committee (HREC). The ethical aspects of this research project have been approved by the HREC of Metro South HREC. This project will be carried out according to the *National Statement on Ethical Conduct in Human Research (2007)*. This statement has been developed to protect the interests of people who agree to participate in human research studies.

# What if I have a question or need to make a complaint?

We have included several contacts for you below. The person you may need to contact will depend on the nature of your query.

If you want any further information concerning this project or if you have any medical problems which may be related to your involvement in the project (for example, any side effects), you can contact the research team at any time:

- *[Local research contact]*, *[Local research contact position]*, telephone: *[telephone]*
- *[Central research contact]*, *[Central research contact position]*, telephone: *[telephone]*

The *[Institute Name]* is committed to research integrity and the ethical conduct of research projects. However, if you wish to discuss the study or with someone not directly involved, particularly in relation to matters concerning policies, information or complaints about the conduct of the study or your rights as a participant, you may contact:

| Reviewing HREC | Metro South |
| --- | --- |
| Contact Person | HREC coordinator |
| Telephone | +61 7 3443 8049 |
| Email | [MSH-Ethics@health.qld.gov.au](mailto:MSH-Ethics@health.qld.gov.au) |
| HREC Reference Number | HREC/2020/QMS/59892 |

| *[Institute]* Governance Office | *[Institute Governance Office]* |
| --- | --- |
| Contact Person | *[Contact Person]* |
| Telephone | *[Telephone]* |
| Email | *[Email]* |

**MASTER Participant Information Sheet/Consent Form for Patients**

| **Title** | Implementation of a nurse-enabled, shared-care follow-up model for early breast cancer survivors |
| --- | --- |
| **Short Title** | The IBIS-Survivorship Study |
| **Coordinating Principal Investigator** | Professor Raymond Chan |
| **Principal Investigator** | *[Institution Principal Investigator]* |

**Declaration by Participant**

I have read the Participant Information Sheet or someone has read it to me in a language that I understand.

I understand the purposes, procedures and risks of the research described in the project.

I understand that during the course of this research records held by public and private hospitals and health services, to include records held by my GP and treating oncologist, may be accessed by my health care providers, Human Research Ethics Committee, research team, and Research Governance Officers to determine my eligibility for participation in this clinical trial and for the purposes of conducting and monitoring the clinical trial and verifying results.

I give permission for my doctors, other health professionals, hospitals or laboratories outside this hospital to release information to *[Name of Institution]* concerning my disease and treatment for the purposes of this project. I understand that such information will remain confidential.

I have had an opportunity to ask questions and I am satisfied with the answers I have received.

I freely agree to participate in this research project as described and understand that I am free to withdraw at any time during the study without affecting my future health care.

I consent to my treating doctor/s being notified of my participation in this study and any clinically relevant information noted by the trial nurse in the conduct of the trial.

I understand that I will be given a signed copy of this document to keep. We may like to ask you to participate in a future related study, or to obtain additional information or clarification related to your participation in this study. Please indicate below whether you are willing to be contacted about any future research studies.

I agree to the research team using, reproducing, and disclosing audio-/video-recordings as explained in the Participant Information Sheet/Consent Form for Patients.

I agree to be audio-/video-recorded and understand that, subject to any constraints requested below, recordings may be used in presentations and publications for educational and research purposes.

**Future Studies**

□ Yes, I agree to be contacted about future research studies

□ No, I do not want to be contacted about future research studies

**30-minute Interview (Optional)**

□ Yes, I agree to be contacted to be invited to participate in the interview

□ No, I do not want to be contacted to be invited to participate in the interview

**Study Results**

□ Yes, I would like to receive a copy of the study results and acknowledge that these will be provided in aggregate (individual results will not be available)

□ No, I do not want a copy of the study results

**Participant Email: ________________________________________________**

**Participant Signature**

| Signature | __________________________________________________ | | Date___________ |
| --- | --- | --- | --- |
| Name of participant (please print) | | __________________________________ Time __________ | |

**Researcher Signature**

| Signature | __________________________________________________ | | Date___________ |
| --- | --- | --- | --- |
| Name of researcher (please print) | | __________________________________ Time __________ | |

***PARTICIPANT CONSENT FORM***

Consent to release of Medicare Benefits Schedule (MBS) and Pharmaceutical Benefits Scheme (PBS) claims information by Services Australia to the Queensland University of Technology for the purposes of the Implementation of a nurse-enabled, shared-care follow-up model for early breast cancer survivors Study

**Important Information (This form is only to be used for participants over 14 years of age)**

Complete this form to request the release of your personal Medicare claims information and your PBS claims information to the Implementation of a nurse-enabled, shared-care follow-up model for early breast cancer survivors Study. Any changes to this form **must** be initialled by the signatory. Incomplete forms may result in the study not being provided with your information.

**Rights and Privacy** (please tick relevant boxes)**:**

I understand that:

⬜ my MBS and PBS information will be disclosed by Services Australia for the purposes of the study.

⬜ the results of this research may be published in articles or journals.

⬜ my name will never be disclosed by Services Australia, used in the study or published.

⬜ my participation in the study is completely voluntary.

⬜ I can withdraw my participation in the study at any time (refer to participant information sheet and withdrawal of consent form) and I do not have to provide a reason for my withdrawal.

**Consent** (please tick relevant boxes)**:**

⬜ I understand the information provided to me about the study I am participating in.

⬜ I have been given the opportunity to ask questions, and any questions I have asked have been answered to my satisfaction.

⬜ I consent to the disclosure by Services Australia of my MBS and PBS information to researchers for the purposes of the study.

**PARTICIPANT DETAILS**

**1.** Mr □ Mrs □ Miss □ Ms □ Other □

Family name: ________________________________ First given name: _________________________

Other given name (s): __________________________

Date of birth: **___ /____/_____**

DD / MM / YYYY

**2.** Medicare card number: ______________________

**3.** Permanent address: _____________________________________________________________

Postal address (if different to above): ________________________________________________

**AUTHORISATION**

**4.** I authorise Services Australia to provide my:

Medicare & PBS claims history

For the period **10/05/2020** to: **10/05/2029** to the Implementation of a nurse-enabled, shared-care follow-up model for early breast cancer survivors Study.

**DECLARATION**

I declare that the information on this form is true and correct.

**5.** Signed: _____________________________ (participant’s signature) Dated: **___ /____/_____**

DD / MM / YYYY March 2020

**Supplementary Material C**

**IBIS-Survivorship Interview Guide – Patients**

1. Can you start by describing your experience in the IBIS-Survivorship Study.
2. Did you have any expectations of the shared care follow-up model for early breast cancer?
3. Let’s talk through your experiences after treatment:
   1. Can you explain your experience of the Nurse-Led Survivorship Clinic For example: How did you find the timing of appointments after treatment had been completed? Did this work for you? Do you have any comments about the content of the clinic (education, goal setting)?
   2. Can you recall you experience with the Survivorship Care Plan?

For example: Did you receive a paper or email copy of your Care Plan? Have you referred to it since the clinic was conducted?

- 1. Can you describe your experience with your GP since you finished treatment?

For example: Did you discuss your cancer treatment and your Survivorship Care Plan with your GP? Have you had follow appointments relating to your cancer since finishing treatment?

- 1. Can you describe your experiences of the follow-up schedule?

For example: Did you feel that you were getting enough support from your specialist team? Did you feel like the timing and amount of appointments after treatment were suitable?

1. Overall, did the care that you experienced after finishing treatment meet your expectations?
2. Can you describe what you felt was the most valuable aspect of care after treatment and why?
3. Is there anything about your care that you would have changed if it were possible?
4. Can you describe anything that was challenging for you that you have not yet mentioned?
5. What information about your breast cancer journey is beneficial to have communicated between the specialist team and your GP, and why?

**IBIS-Survivorship Interview Guide – Healthcare Providers**

1. Can you tell me your initial thoughts on shared-care follow-up for people with early breast cancer, in general.
2. Tell me about your experience of implementing the IBIS-Survivorship care model in your health service. *Prompt regarding components below*:
   1. Nurse-led consultation
   2. Survivorship Care Plan
   3. GP case conference
   4. Shared care follow-up
3. Tell me whether you think the implementation of IBIS-Survivorship was successful in your clinic? Why or why not? What aspects?
4. Can you tell me any barriers to implementing the IBIS-Survivorship model?
5. Do you think there are aspects of the IBIS-Survivorship model that are effective for patients, healthcare providers or health service capacity?
6. Have you implemented any elements of IBIS-Survivorship or shared care models since recruitment finished?
