# Supplementary material for: Implementation and effectiveness of a nurse-enabled, shared-care follow-up model for early breast cancer survivors (The IBIS-Survivorship Study): protocol for a stepped-wedge cluster randomised trial
Source: BMJ Open. 2025 Jun 19;15(6):e103341. doi: 10.1136/bmjopen-2025-103341 (PMC12182111; doi:10.1136/bmjopen-2025-103341)
Supplement: Supplementary file 2 [file bmjopen-2025-103341.reviewer_comments.pdf]

PEER REVIEW HISTORY

BMJ Open publishes all reviews undertaken for accepted manuscripts. Reviewers are asked to complete a checklist review form and are provided with free text boxes to elaborate on their assessment. These free text comments are reproduced below.

ARTICLE DETAILS

Title (Provisional)

Implementation and effectiveness of a nurse-enabled, shared-care follow-up model for early breast cancer survivors (The IBIS-Survivorship Study): Protocol for a stepped-wedge cluster randomised trial

Authors

Chan, Raymond J; Crawford-Williams, Fiona; Koczwara, Bogda; Mann, G. Bruce; Eakin, Elizabeth; Turner, Jane; Krishnasamy, Meinir; Collins, Louisa G; Skerman, Helen; Hemming, Karla; Hart , Nicolas H; Emery, Jon; Crowe, Bethany; Patford, Kerry; Mahony, Jane; Kristunas, Caroline; Blanchard, Gillian; Healey, Laura; Sanmugarajah, Jasotha; Liang, Rhea; Craft, Paul; Shorthouse, Amy; McCaffery, Ruth; Azer, Mary; Yates, Patsy

---

This article was not externally reviewed at BMJ Open. Protocols that have been independently assessed prior to submission to BMJ Open are usually fast-tracked to publication on the grounds that further substantial changes will not be possible. This independent assessment will usually be external, independent review for both a substantial grant award from a non-commercial or government funder and ethics approval.
